# Enzymatic Synthesis of Protein Hydrolysates From Animal Proteins: Exploring Microbial Peptidases

**DOI:** 10.3389/fmicb.2018.00735

**Published:** 2018-04-16

**Authors:** Ronivaldo Rodrigues da Silva

**Affiliations:** Instituto de Biociências, Letras e Ciências Exatas, Universidade Estadual Paulista Júlio de Mesquita Filho (UNESP), São José do Rio Preto, Brazil

**Keywords:** collagen, keratin, protease, protein, proteolysis

## Microbial peptidases in biotechnology

Proteins, components essential to all organisms, are integrated in cellular structures and perform specific functions as in the case of hormones, antibodies, and enzymes. Enzymes are biocatalysts that are responsible for biochemical transformations fundamental to the functioning of all cellular metabolism (Silva, 2017).

Among the enzymes, peptidases are capable of cleaving peptide bonds in proteins and peptides. These enzymes are encoded by about 2% of the genes in all kinds of organisms (MEROPS—the Peptidase Database)^1^.

In addition to their important physiological functions, peptidases are widely exploited for their application in various industrial segments and basic research (Fang et al., 2013; Gopinath et al., 2015; da Silva et al., 2016, 2017a,b; Silva, 2017, 2018a; Silva et al., 2017c). Due to their versatility, some peptidases are capable of cleaving keratinous residues and collagen, and have garnered great interest in the degradation of animal proteins that are mainly found as disposable residues from industrial activity (Gopinath et al., 2015; Lange et al., 2016; Verma et al., 2017).

Thus, the use of these enzymes represents an important strategy in biotechnology because it promotes the usage of animal residues such as collagen and keratin, and adds economic value to the products derived from these compounds. This is particularly true with respect to the production of protein hydrolysates, which synthesizes peptides that are important for the various biological functions in the body (Bhat and Kumar, 2015), and can be applied as animal supplements or biostimulant in order to grow vegetables (Silva, 2017).

Exploring microbial diversity for peptidase production appears to be a promising biotechnological resource for studies researching the synthesis of protein hydrolysates. However, the concrete advances of these investigations need to be determined along with the future prospects and expected results for this biotechnological area.

To date, numerous research groups have used microbial peptidases to synthesize peptides and prospect bacterial and fungal peptidases (Gousterova et al., 2005; Bhaskar et al., 2007; Fang et al., 2013; Gopinath et al., 2015; Silva, 2017; Verma et al., 2017). It has advanced the application of protein hydrolysates and facilitated a promising future for this field.

## Biologically functional hydrolysates

Enzymatic proteolysis is the most common way to synthesize protein hydrolysates (Bhat and Kumar, 2015). Based on the catalytic properties of the peptidase with respect to substrate specificity, different oligopeptides can be obtained via enzyme-substrate interaction (Silva, 2018b). Thus, several investigations have been performed to determine new enzymes that act with different specificities under a variety of proteins.

In the last few years, growing interest has emerged toward plant peptidases and their application in peptide synthesis. However, digestive and microbial peptidases are the major enzymes that are used in this process. Among these biocatalysts, digestive enzymes like pepsin, trypsin, and chymotrypsin, and bacterial and fungal peptidases like alcalase, neutrase, flavourzyme, and other new peptidases derived by prospecting bacteria and fungi (Silva, 2017) are the most common enzymes employed for the synthesis of protein hydrolysates (De Castro and Sato, 2014; Bhat and Kumar, 2015).

**Figure 1:**
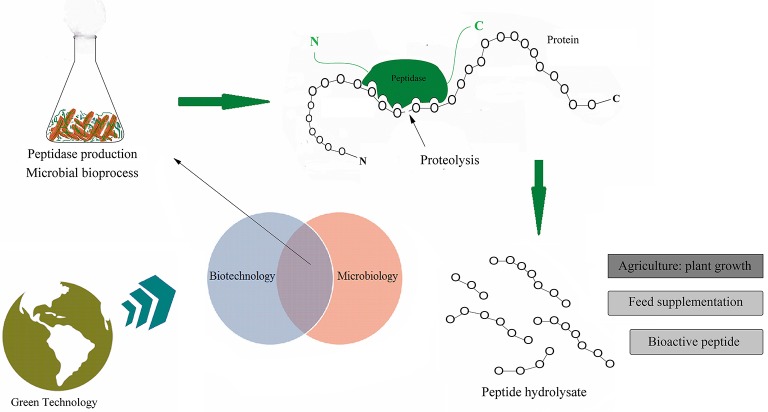
Peptidases as a promising biocatalyst in sustainable processes. By exploring the microbial biodiversity the peptidases have been investigated. The illustration shows a simple scheme of production of these enzymes from microorganisms and their action in the decomposition of animal proteins and production of peptide hydrolysate.

The increased interest in protein hydrolysates has promoted important advances in this field of study. It has been particularly driven by research in industrial microbiology/enzyme technology, which is largely dedicated to prospecting new microbial peptidases. It constitutes a sustainable technological strategy that obtains peptides at reduced production costs (Figure 1).

As the central theme of this opinion article, I focused on enzymatic synthesis of protein hydrolysate derived from animal proteins and its use as a supplement to animal diets and as a biostimulant in plant cultivation.

## Protein hydrolysates in agriculture and supplements for animal diets

The availability of minerals in the soil is an important factor that determines plant growth. Fertilizers have been used for years to ensure good harvests in agriculture and to correct issues such as low fertility (Bhardwaj et al., 2014).

Chemical fertilizers are compounds rich in nitrogen, phosphorus, and potassium in defined amounts. With the increasing demand for food due to the progressively increasing world population, agricultural processes use inorganic fertilizers to meet market demands (Bhardwaj et al., 2014).

However, the frequent use of chemical agents has contributed to an increase in soil and water contamination (Bhardwaj et al., 2014; Santi et al., 2017). Alternative compounds, capable of improving soil fertility, reduce production costs in order to reduce the consumer prices of vegetables products have been much expected.

As advances in sustainable technologies, biological strategies have been applied to reduce the use of inorganic fertilizers (Bhardwaj et al., 2014; Subbarao et al., 2015; Santi et al., 2017). In general, the use of microbial-based fertilizer, which employs microorganisms to improve soil fertility and nutrient uptake, has been widely accepted for use in agriculture (Bhardwaj et al., 2014). Another growing trend has been the promising effect of protein hydrolysate on plant growth (Santi et al., 2017; Verma et al., 2017). As previously mentioned, the application of these hydrolysates has advantage to favor the use of residues derived from animal proteins.

Protein hydrolysates have gained attention for their use in agriculture as they have been shown to be efficient in improving the fertility of the soil. The critical evaluation of this field of study demonstrates advances in plant biostimulants. The global biostimulant market has expected to grow at a value estimated to reach $ 3.79 billion by 2023 (https://www.reuters.com/brandfeatures/venture-capital/article?id=13042). In this field, some attention has been devoted to hydrolyzate-based proteins with promising results.

Plants treated with protein hydrolysates presented better growth than plants grown using inorganic nitrogen. Santi et al. (2017) demonstrated a 7-fold increase in length and a 1.5-fold increase in root surface area when comparing protein hydrolysate- treated maize and non-treated plants. Maize roots grown using protein hydrolysates as biostimulant also showed an increase in K, Zn, Cu, and Mn when compared to inorganic fertilizers.

The application of these compounds as a biostimulant has been shown to be very important for the growth of plants, resulting in increased growth of root and leaf biomass. Additionally, it acts as a source of nutrients for soil microorganisms, thus improving biological activity and nutrient cycling (Santi et al., 2017).

Subbarao et al. (2015) showed improvement in the productivity of different crop species (paddy rice, finger millets, cowpeas, and radish) when they were treated with protein hydrolysates (protein from skin and hair wastes). The study revealed that the use of hydrolysates in the soil exerted better biostimulating activity in plants than foliar treatment. However, the cultivation of the plants under hydrolysate treatment in both cases was better than the control experiment (without treatment). The authors observed improvements in several aspects of the plant, such as root and shoot length, leaf area, total chlorophyll content, and photosynthetic rate.

As demonstrated in some studies, proteolytic enzymes have been prospected to successfully act on different proteins such as collagen and keratin, which demonstrates the real opportunity in using these biocatalysts as strategic tools for green technology in order to reduce the use of chemical agents and to utilize these hydrolysates as plant biostimulant. In addition, according to the nutritional value of hydrolysates derived from animal proteins, these compounds have their application also directed to animal feed supplementation, conferring improved animal growth in cattle, chicken, and sheep, among others (Verma et al., 2017; Silva, 2018b).

Ichida et al. (2001) described a successful keratin degradation process using *Bacillus licheniformis* and a *Streptomyces* sp., and its potential application in animal feed and plant growth. Fang et al. (2013) also reported wool keratin degradation by *Stenotrophomonas maltophilia*. In this investigation, the authors observed the presence of 17 different amino acids on the hydrolysate, where a large amount of an essential amino acid (phenylalanine) was observed, reaching 92.67 mg/L after 4 days of fermentation. The high amino acid content suggests hydrolysate usage as a potential nutritional additive.

In another report, Veselá and Friedrich (2009) exhibited the potential application of protein hydrolysate as a foliar biostimulant. The authors obtained the hydrolysate via the enzymatic hydrolysis of bovine hooves and horns using keratinase from *Paecilomyces marquandii*. On hydrolysate was detected the presence of protein, peptides and 18 different free amino acids, in which a large amount of nonpolar neutral, basic, and sulfur amino acids was observed. The high amino acid content released from the protein degradation formed an interesting nutritional supplement, which it is easier to absorb because of the presence of free amino acids.

The animal-based food industry (chickens, cattle, pig, and fish) generates large amounts of low-acceptability residues such as skin, hair, viscera, blood, and feathers. These animal residues serve as raw materials in the synthesis of protein hydrolysates for application as plant biostimulant and nutritional additives (Silva, 2017). Therefore, it is possible that several industrial segments generate protein byproducts that can be redirected toward enzymatic processing and generation of protein hydrolysates.

Bhaskar et al. (2008) reported the enzymatic hydrolysis of visceral waste protein from *Catla catla* using the Alcalase® enzyme from *Bacillus licheniformis*. The protein hydrolysate, containing large amounts of arginine, asparagine/aspartate, glutamine/glutamate, glycine, alanine, and proline/hydroxyproline, demonstrated its potential for use in fish diet. Kechaou et al. (2009) also reported a protein hydrolysate obtained from fish viscera (*Sepia officinalis* and *Sardina pilchardus*) using commercial enzymes from microorganisms, such as Alcalase® and Flavourzyme® (Novozymes/DK). According to the free amino acid composition, the results also indicated the potential use of the hydrolysate as a supplement for animal diet.

Table 1 shows some examples of protein hydrolysates from different animal proteins and their potential application as plant biostimulants and animal feed supplementation.

**Table 1:** Examples of protein hydrolysate derived from different animal protein and their potential application.

**Microorganism**	**Protein source**	**Potential application**	**References**
Keratinase from *Paecilomyces marquandii*	Bovine hooves and horns	Foliar fertilizer	Veselá and Friedrich, 2009
*Bacillus licheniformis* and *Streptomyces* sp.	Poultry waste	Biofertilizer	Ichida et al., 2001
*Stenotrophomonas maltophilia*	Wool keratin	Biofertilizer and animal nutrition	Fang et al., 2013
*Thermoactinomyces* strains	Sheep skin and wool	Biofertilizer and animal nutrition	Gousterova et al., 2005
Alcalase from *Bacillus licheniformis*	Visceral waste from fish	Additive in diet fish	Bhaskar et al., 2008
Alcalase (*B. licheniformis*) and Flavourzyme (*Aspergillus oryzae*)	Visceral waste from fishies	Supplement in animal diet	Kechaou et al., 2009
Fungal Protease P	Sheep visceral mass (stomach and intestines)	Nutritional supplement in animal diet	Bhaskar et al., 2007

As reported in this opinion article, the high amounts of free amino acids and oligopeptides released from animal protein hydrolysis are of great interest for several applications in agriculture and livestock farming. Notably, the application of microbial enzymes has revolutionized various industrial sectors. In order to understand the advances in this area of study, the potential of the enzyme-based products has been clearly demonstrated in this article. Hydrolysis of animal proteins and their potential in animal nutrition and plant growth are examples of advances in enzymatic technology in favor of sustainable anthropogenic activity.

To date, the research proves the applicability of protein hydrolysates derived from animal residues. It presents an opportunity to reduce the uncontrolled use of inorganic fertilizers, which can be completely or partial substituted with peptide hydrolysates (additive to other biostimulants and biofertilizers) in agriculture.

## Future directions

Hydrolysate production via the enzymatic degradation of protein animal is a well-accepted and promising method that can solve the inappropriate disposal issues of animal residues in the environment, while adding to the economic value of this organic matter.

Therefore, industrial activity from several sectors generate protein byproducts that can be redirected toward enzymatic processing and generation of protein hydrolysates. This reinforces the need to prospect for new enzymes and strengthens the requirement for technological advances aimed at reducing the disposal of industrial waste and adding economic value to its own byproducts.

The improvement of further application methodologies, including technologies for the production of protein hydrolysates and peptidases prospecting, are fundamental in establishing progress with respect to this biotechnological segment.

## Author contributions

The author confirms being the sole contributor of this work and approved it for publication.

### Conflict of interest statement

The author declares that the research was conducted in the absence of any commercial or financial relationships that could be construed as a potential conflict of interest. The reviewer SR and handling Editor declared their shared affiliation.

## Footnotes

^1^MEROPS – The Peptidase Database. Available online at: https://www.ebi.ac.uk/merops/about/peptidases_faq.shtml (Accessed 9, 2017).
